# Test, Treat, Track, Test, and Treat Active Surveillance toward Elimination of Schistosomiasis: A Feasibility Study

**DOI:** 10.4269/ajtmh.20-0156

**Published:** 2020-07-13

**Authors:** Reda M. R. Ramzy, Amal Rabiee, Khaled M. Abd Elaziz, Carl H. Campbell, Nupur Kittur, Daniel G. Colley, Ayat A. Haggag

**Affiliations:** 1 National Nutrition Institute, General Organization for Teaching Hospitals and Institutes, Cairo, Egypt;; 2 Ministry of Health and Population, Cairo, Egypt;; 3 Department of Community, Environmental and Occupational Medicine, Faculty of Medicine, Ain Shams University, Cairo, Egypt;; 4 Center for Tropical and Emerging Global Diseases, University of Georgia, Athens, Georgia;; 5 Department of Microbiology, University of Georgia, Athens, Georgia

## Abstract

We assessed the feasibility of using a test, treat, track, test, and treat (5T) active surveillance strategy to identify and treat individuals with schistosomiasis in three very low-prevalence villages in Kafr El Sheikh Governorate, Egypt. Primary index cases (PICs) were identified using the point-of-care circulating cathodic antigen (POC-CCA) assay in schools, in rural health units (retesting individuals with positive Kato–Katz examinations over the previous 6 months), and at potential water transmission sites identified by PICs and field observations. Primary cases identified potential second-generation cases—people with whom they shared water activities—who were then tracked, tested, and treated if infected. Those sharing water activities with second-generation cases were also tested. The yield of PICs from the three venues were 128 of 3,576 schoolchildren (3.6%), 42 of 696 in rural health units (6.0%), and 83 of 1,156 at water contact sites (7.2%). There were 118 second- and 19 third-generation cases identified. Persons testing positive were treated with praziquantel. Of 388 persons treated, 368 (94.8%) had posttreatment POC-CCA tests 3–4 weeks after treatment, and 81.8% (301) became negative. The 67 persons remaining positive had negative results after a second treatment. Therefore, all those found positive, treated, and followed up were negative following one or two treatments. Analysis of efforts as expressed in person-hours indicates that 4,459 person-hours were required for these 5T activities, with nearly 65% of that time spent carrying out interviews, treatments, and evaluations following treatment. The 5T strategy appears feasible and acceptable as programs move toward elimination.

## INTRODUCTION

Human schistosomiasis or Bilharzia is a neglected tropical disease (NTD) caused by blood flukes of the genus *Schistosoma*, currently endemic in 76 countries worldwide and has infected approximately 229.2 million people in 2018.^1^ Two forms of the disease are found in Africa: *Schistosoma haematobium* causes the urogenital form of the disease and *Schistosoma mansoni* causes the intestinal form of the disease. Infected people are mainly school-age children in rural areas as well as workers (farmers and fishermen) in contact with freshwater bodies infested with intermediate host snails.^2^

The WHO recommends preventive chemotherapy using anthelmintic drugs, either alone or in combination, as a public health measure for control/elimination of schistosomiasis in humans. Preventive chemotherapy can be implemented in three ways: 1) mass drug administration (MDA) when the whole population of a predefined geographical area is treated irrespective of clinical status, 2) targeted chemotherapy when only specific risk groups are treated; and 3) selective chemotherapy which screens patients and subsequently treats according to clinical status.^3^ Implementation of intensive control measures for several decades has resulted in significant reduction in the prevalence and morbidity of schistosomiasis in countries where the disease was highly endemic in the past, including China, Brazil, and Egypt.^4^ As shown in parts of Egypt and China, if carried out for decades, control interventions based on MDA can achieve very low levels of prevalence. At that point, MDA cost per case will be high, and at least 90% of people treated will be uninfected, leading to massive overuse of drugs, and resources, and treatment fatigue.

Selective chemotherapy or testing, treatment, and surveillance provide a more cost-effective approach for chasing low numbers of remaining infected cases. For such an approach to be successful, every suspected *S. mansoni* case should be tested using a reliable rapid test and confirmed cases should be treated using praziquantel (PZQ). Then, the disease should be tracked through active epidemiological surveillance. Note that in 2012, the WHO initiated a comparable malaria “T3”: test, treat, and track” as a useful tool for eliminating malaria deaths and eventually eradicating the disease.^5^

In this study, we evaluated the feasibility and effectiveness of an alternative approach for MDA in three communities, with very low *S. mansoni* prevalence in the Nile Delta region of Egypt. This strategy, termed as the “test, treat, track, test, and treat” (5T), is based on testing for *S. mansoni* infection using the urine-based point-of-care circulating cathodic antigen (POC-CCA) test, and treatment of confirmed cases using PZQ. They were tracked, tested with another POC-CCA assay after 3–4 weeks, and retreated, if found POC-CCA positive.

## MATERIALS AND METHODS

### Ethics statement.

The Ethics Review Committee of the Ministry of Health and Population reviewed and approved the study protocol (May 18, 2019 dated March 21, 2019). Children were enrolled in the study after obtaining informed consent from their parents/guardians. Likewise, all enrolled adults provided informed consent. The work included only noninvasive collections of urine specimens. Providing a urine sample was taken as a child’s assent.

### Study area.

The study was conducted in three villages each located in Desouk, Al Riad or Sidy Salem district, Kafr El Sheikh Governorate. Each village has a population of approximately 6,000 people. The study villages were selected based on records in files at the Ministry of Health and population indicating low to very low *S. mansoni* prevalence (≈10% by POC-CCA assay).

### Study participants.

Schoolchildren (6–14 years), attending three schools in the study villages, were included in this study. All POC-CCA assay–positive subjects (scored as 1+, 2+, and 3+), identified during the school survey, were included in the study as primary index cases (PICs). Because we have previously shown that in areas of very low prevalence, trace and sometimes 1+ band readings of POC-CCA are likely to represent false-positive readings,^6^ subjects with POC-CCA scored as 1+, 2+, and 3+ were considered infected in the current work. This level of the POC-CCA reading (1+ and above) was decided as the positive cutoff for this study because the object of a 5T approach is to identify as many of those who are likely to be positive, so they can be treated and tracked. Furthermore, schistosomiasis-infected subjects recorded in the rural health units, based on Kato–Katz stool examination, during the 6 months before this study (October 2018–March 2019), were examined by POC-CCA assay, and all positives were included as PICs. In addition, POC-CCA assay–positive individuals, identified during direct observations of water contact sites, were included in the study as PICs.

All identified PICs were interviewed using a validated questionnaire to determine where they had contact with water and to determine the other individuals who frequented those potential water contact sites. Accordingly, individuals sharing water activities with PICs were identified and examined for schistosomiasis by using the POC-CCA assay. All POC-CCA assay–positive subjects (scored as 1+, 2+, and 3+) were included in the study as secondary index cases (SICs). Similarly, SICs were interviewed to identify other individuals sharing water activities with them, and POC-CCA assay–positive individuals from the latter group were included as the second-generation of SICs.

### Assessment of water contact sites.

Potential water contact sites were identified based on interviews with PICs and SICs, as well as by direct observations of individuals who then participated in the study. Assessments of potential water contact sites were carried out during the last week (i.e., 7 days) of each month during March–June 2019. For each potential water contact site, direct observation was carried out in two shifts, from 7 am to 12.30 pm and from 12.30 pm to 5–6 pm. For each potential water contact site, the number of individuals entering the water, those contacting the water site for the first time, and the type of water activity were recorded. At the end of each observational week, recorded individuals were approached and asked to participate in the study. Those who consented (for children, consent was sought from parents or guardians at their homes), were assigned a coding number and enrolled in the study.

### Point-of-care circulating cathodic antigen assay.

The POC-CCA test (batch number 181116119, Ex. 11/2020), was performed according to the manufacturer’s instruction (Rapid Medical Diagnostics, Pretoria, South Africa). In reading the POC-CCA results, we used grades 1+ (intensity of the reactive band equal to the control band), 2+, and 3+ for scoring positive reactions. In this setting, trace reactions (fainter than the control band) was considered negative. The POC-CCA test was performed in three venues: primary schools, rural health units, and potential water contact sites.

### Treatment of infected participants.

All subjects with positive POC-CCA test results were treated using a standard PZQ dose (directly observed treatment with a single dose of 40 mg/kg body weight). They were retested about 3–4 weeks after their initial PZQ dose and retreated, if they remained POC-CCA positive (not cured).

### Recording information about the 5T process.

Recorded information about the 5T process included the time taken for various steps, the level of difficulty of each step, cost of local transportation, suggestions for increasing efficiency, yield in terms of individuals tested, and individuals testing positive using various approaches to identify potentially infected individuals, and barriers to implementing the test and treat strategy. These data were recorded on validated questionnaires by technicians and health workers involved in the study. For example, the level of difficulty of each step was reported using a scale of 1–5 (1, very easy; 2, easy; 3, moderate; 4, difficult; and 5, very difficult).

### Statistical analysis.

Data were checked for completeness and consistency. Data entry was carried out on Microsoft Excel database spreadsheet. Quantitative data were summarized by mean and SD, whereas qualitative data were summarized by frequencies and percentages. Data analysis was calculated with IBM SPSS statistics for Windows version 23 (IBM Corp., Armonk, NY). The chi-square test was used in analysis of this study. A “*P*-value” of less than 0.05 was considered statistically significant.

## RESULTS

### Identification of PICs by different venues.

#### School survey.

**Figure 1.**
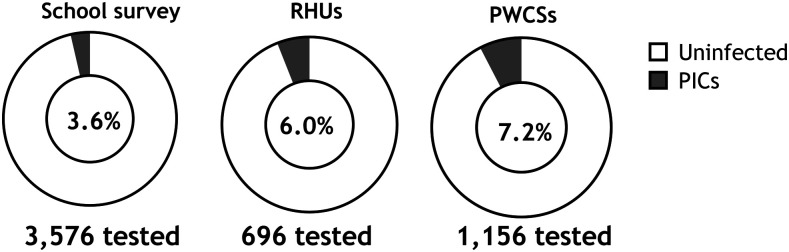
Yield of primary index cases (PICs) as identified by three different case detection venues in low-prevalence *Schistosoma mansoni* areas in Egypt. PWCSs = potential water contact sites; RHUs = rural health units.

A total of 3,576 schoolchildren (6–15 years) were examined in the three villages of the study (Table 1, Figure 1). Of these, 1,753 children (49%) and 1,823 children (51%) were males and females, respectively. Of the schoolchildren tested, 3.6% (*n* = 128) were POC-CCA positive and included as PICs (Figure 1). The school survey revealed that the percentage of identifying PICs in Desouk (1.6%) was lower than that in Al Riad (5.1%) and Sidy Salem (4.4%) villages (*X*^2^ = 22.8, *P* = 0.001; *X*^2^ = 17.2, *P* = 0.001, respectively).

**Table 1:** Number of PICs as determined in three different case detection venues in low-prevalence *Schistosoma mansoni* areas in Egypt

Study site	School survey (children aged 6–15 years)		Screening at rural health units		Surveillance at potential water contact sites	
	Examined, *n*	PICs,* *n* (%)	Examined, *n*	PICs, *n* (%)	Examined, *n*	PICs,† *n* (%)
Desouk	1,280	20 (1.6)	381	25 (6.6)	393	26 (6.6)
Al Riad	1,021	52 (5.1)	–	–	413	19 (4.6)
Sidy Salem	1,275	56 (4.4)	315	17 (5.4)	350	38 (10.9)
Total	3,576	128 (3.6)	696	42 (6.0)	1,156	83 (7.2)

PIC = primary index cases.

* Number of PICs identified in Desouk schools was lower than that identified in Al Riad and Sidy Salem villages (*X*^2^ = 22.8, *P* = 0.001; *X*^2^ = 17.2, *P* = 0.001, respectively).

† Number of PICs identified in Sidy Salem potential water contact site was significantly higher than that identified in Desouk and Al Riad villages (*X*^2^ = 4.3, *P* = 0.03 and *X*^2^ = 10.8, *P* = 0.001, respectively).

#### Rural health units.

Throughout the study, the rural health unit in Al Riad was under renovation, and so this part of the study was not conducted in this village. During the study period, 696 subjects were referred to the rural health units in the other two villages (Desouk and Sidy Salem villages) for examination of *S. mansoni* infection (Table 1, Figure 1). Of these, 381 subjects (54.7%) were from Desouk village, their age ranged between 4 and 66 (mean ± SD; 35.9 ± 13.9) years, and 224 (58.8%) were males and 157 (41.2%) females. Whereas, 315 subjects (45.3%) were referred to the rural health unit in Sidy Salem village, their age ranged between 6 and 76 (27.6 ± 15.7) years, and 195 (61.9%) were males and 120 (38.1%) females. Overall, 42 subjects (6%) were POC-CCA positive in rural health units and included as PICs (Figure 1). There was no difference in identifying PICs between the two villages.

#### Potential water contact sites.

Assessment of the potential water contact sites revealed that 1,156 participants were in contact with water at least one time and were POC-CCA tested on their first contact. Of these, 393 (34.0%), 413 (35.7%), and 350 (30.3%) were from Desouk, Al Riad, and Sidy Salem villages, respectively (Table 1). Overall, 83 subjects (7.2%) were POC-CCA positive in assessment of potential water contact sites and included as PICs (Figure 1). Assessment of potential water contact sites revealed that the percentage of identifying PICs was significantly higher in Sidy Salem (10.9%) than that in Desouk (6.6%) and Al Riad (4.6%) villages (*X*^2^ = 4.3, *P* = 0.03 and *X*^2^ = 10.8, *P* = 0.001, respectively).

In general, the proportion of PICs identified as a result of the school survey (3.6%) was significantly lower than the ratio of identifying PICs at rural health units (6%) (*X*^2^ = 8.7, *P* = 0.003) and potential water contact sites (7.2%) (*X*^2^ = 30.9, *P* = 0.0001).

### Identification of SICs and second-generation SICs by different venues.

**Figure 2.**
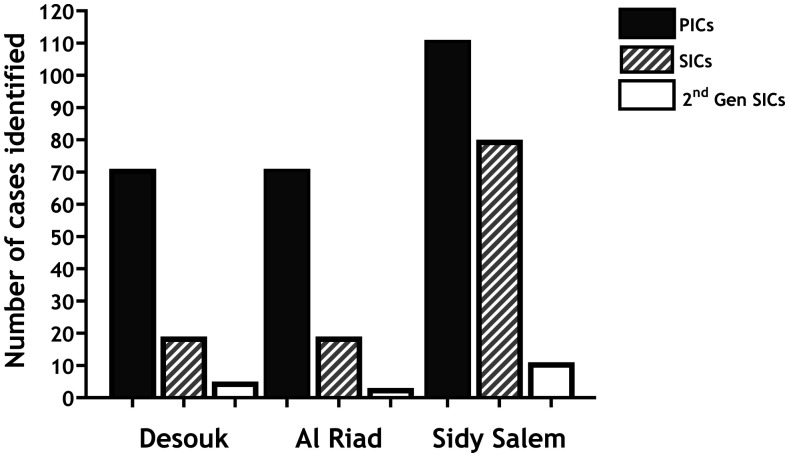
Number of primary index cases (PICs), secondary index cases (SICs) and second-generation SICs as presented by village. The proportion of PICs and SICs identified in Sidy Salem was significantly higher than that in Desouk and Al Riad (*X*^2^ = 11.5, *P* = 0.0007; *X*^2^ = 42.7, *P* = 0.0001, respectively). The proportion of second-generation SICs identified in Sidy Salem was higher than that in Al Riad (*X*^2^ = 4.4, *P* = 0.03) but not than that in Desouk.

Of the initially tested 5,428, the study identified a total of 390 infected subjects (7.2%), categorized as PICs (64.8%), SICs (30.3%), and second-generation SICs (4.9%). Their age ranged between 4 and 70 (26.6 ± 16.9) years, and 270 (69.2%) were males and 120 (30.8%) females. They included 95 subjects (24.4%) from Desouk village, 93 (23.8%) from Al Riad, and 202 (51.8%) from Sidy Salem village (Figure 2). The POC-CCA readings ranged between 1+ (254 subjects; 65.1%), 2+ (102 subjects; 26.2%), and 3+ (34 subjects; 8.7%). The percentage of PICs and SICs identified in Sidy Salem was significantly higher than that in Desouk and Al Riad (*X*^2^ = 11.5, *P* = 0.0007; *X*^2^ = 42.7, *P* = 0.0001, respectively). In addition, the percentage of SIC second-generation identified in Sidy Salem was higher that than in Al Riad (*X*^2^ = 4.4, *P* = 0.03) but not than that in Desouk.

### Assessment of potential water contact sites.

Potential water contact sites were assessed over 1 week of each of 4 months of the study duration. In Desouk village, the main recorded activities were agriculture practices (irrigation, surface irrigation for rice seedlings, etc.) and fishing (46.2%), washing utensils and clothes (39.2%), and swimming and recreation (14.6%). In Al Riad village, proportion recorded in these three activities was 69%, 15.5%, and 15.5%, respectively, whereas in Sidy Salem village, it was 70.6%, 16.8%, and 12.6%, respectively.

### Treatment of identified PICs and SICs.

All subjects tested POC-CCA positive were offered treatment with a standard PZQ dose (directly observed treatment with a single dose of 40 mg/kg body weight) and followed up after 3–4 weeks. Table 2 shows the data of PICs and SICs treatment with PZQ and follow-up with POC-CCA at 3–4 weeks after treatment. Of the 253 PICs, 251 subjects (99.2%) were treated, although two individuals refused treatment. At the time of the follow-up, a lower percentage (95.6%) (*n* = 240) of treated subjects (*X*^2^ = 6.4, *P* = 0.01) were available for evaluation. In those evaluated, the cure rate reached 80.0% (*n* = 192) and those who remained POC-CCA positive all turned negative (cured) after a second dose of PZQ. Similarly, 94.0% and 89.5% of the treated SICs and second-generation SICs were available for posttreatment follow-up evaluation, and the cure rate reached 85.6% and 82.3%, respectively. The overall cure rate reached 81.7% after a single PZQ dose and 100% after a second dose. There was no difference in the cure rate (turning POC-CCA negative) between the three study categories.

**Table 2:** Number of PICs and SICs treated with praziquantel and followed up with POC-CCA at 3–4 weeks after treatment

Category of cases	Identified, *n*	Treated, *n*	Treatment outcome, *n* (%)		
			Followed up	Cured after single `dose	Cured after two doses
PICs	253	251*	240 (95.6)	192 (80.0)	48 (20.0)
SICs	118	118	111 (94.0)	95 (85.6)	16 (14.4)
Second-generation SICs	19	19	17 (89.5)	14 (82.3)	3 (17.7)
Total	390	388	368† (94.8)	301 (81.7)	67 (18.3)

PIC = primary index cases; POC-CCA = point-of-care circulating cathodic antigen; SIC = secondary index cases. There was no difference in the cure rate (turning POC-CCA negative) between the three case categories.

* Two PICs refused treatment.

† Twenty subjects were not available for posttreatment evaluation.

### Assessment of the 5T process.

**Figure 3.**
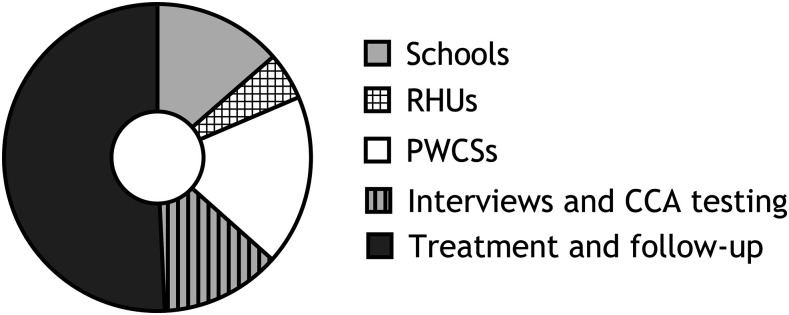
Percentage of person-hours spent in pursuing the test, treat, track, test, and treat strategy in three different case detection venues in low-prevalence *Schistosoma mansoni* areas in Egypt, including testing and interviews, treatment, and follow-up after treatment. Half the effort was for treating primary index cases and secondary index cases and follow-up evaluation. RHUs = rural health units; PWCSs = potential water contact sites.

The overall efforts for pursuing the different 5T tasks added up to 4,459 person-hours. The least amount of effort (222 person-hours, 5%) was spent at rural health units followed by efforts at schools (605 person-hours, 13.6%), and then 565 person-hours (12.7%) for POC-CCA testing and interviews, followed by effort for assessment of potential water contact sites (804 person-hours, 18%), whereas the highest number of person-hours (2,263, 50.8%) was needed for treatment and follow-up evaluations (Figure 3, Table 3). Among the study villages, there was no difference between the percentage of person-hours dedicated for activities at schools and at rural health units, assessment of potential water sites, and interviews and POC-CCA testing. However, the percentage of person-hours dedicated for treatment and follow-up evaluations was significantly higher in Sidy Salem villages that that in the other two study villages (*X*^2^ = 8.552; *P*= 0.0035 and *X*^2^ = 8.024; *P*= 0.0046, for Desouk and Al Riad, respectively).

**Table 3:** Efforts dedicated for pursuing different test, treat, track, test, and treat activities as expressed in person-hours

Study site	Activity in person-hour (%)					Total
	School survey	Identification of primary index cases at rural health units	Assessment of potential water sites	Circulating cathodic antigen testing and interviews	Treatment and follow-up	
Desouk	192 (4.3)	99 (2.2)	273 (6.1)	176 (3.9)	703 (15.8)	1,443 (32.4)
Al Riad	197 (4.4)	–	249 (5.6)	177 (4.0)	710 (15.9)	1,333 (29.9)
Sidy Salem	216 (4.8)	123 (2.8)	282 (6.3)	212 (4.8)	850 (19.1)	1,683 (37.7)
Total	605 (13.6)	222 (5.0)	804 (18.0)	565 (12.7)	2,263 (50.8)	4,459 (100)

Regarding activities at schools, rural health units, and assessment of potential water sites, there was no difference between the percentage of efforts dedicated in each village. A significantly higher percentage of person-hours was spent to carry out interviews, treatment, and follow-up evaluation in Sidy Salem village than Desouk and Al Riad (*X*2 = 9.5, *P* = 0.008) villages.

The overall cost of transportation within the study area (study village and its surroundings) was estimated as US$1,445.50. Of this, 29.1% (US$422.00) was spent in Desouk village, 24.5% (US$354.50) in Al Riad, and 46.3% (US$669.00) in Sidy Salem village. The estimated cost of transportation was significantly higher in Sidy Salem village than Desouk (*X*^2^ = 90.974; *P*= 0.0001) and Al Riad village (*X*^2^ = 150.094; *P*= 0.0001).

The level of difficulty of each step was reported by each of 24 persons (technicians and health workers) who were involved in conducting the study. On a scale of 1–5 (1 = very easy and 5 = very difficult), the school survey was rated very easy and easy by 70.8% and 29.2%, respectively. Working at rural health units, in Desouk and Sidy Salem combined, was rated very easy and easy by 75% and 25%, respectively. Assessment of potential water contact sites was rated easy and difficult, each by 50% of the reporting persons. Treatment of infected subjects was rated very easy, easy, and moderate by 20.8%, 41.7%, and 37.5%, respectively. Posttreatment follow-up evaluation was rated moderate, difficult, and very difficult by 33.3%, 37.5%, and 29.2%, respectively.

## DISCUSSION

The present study aimed to assess the feasibility of a new initiative T5: test, treat, track, test, and treat of human schistosomiasis *mansoni*. The strategy is based on active surveillance for identifying *S. mansoni*–infected cases through three venues: primary schools, rural health units, and potential water contact sites. *Schistosoma mansoni* infection was determined using the POC-CCA test, and subjects with POC-CCA scored as 1+, 2+, and 3+ were considered infected in the current work. Infected cases were treated with a standard dose of PZQ and followed up with another POC-CCA assay after 3–4 weeks. The extensive active surveillance in the study villages, which had been under schistosomiasis control for many decades, confirmed their low *S. mansoni* prevalence (≤ 10% by POC-CCA assay), based on examination of schoolchildren,^7^ as the overall infection prevalence ranged between 3.6% (school survey) and 7.2% (assessment of potential water contact sites).

Comparison between the three study venues indicated that both the potential water contact site and rural health unit approaches identified larger numbers/percentages of *S. mansoni* infections than primary school surveys. This is probably because for many decades, schoolchildren in these schools had received annual mass treatment for schistosomiasis using standard dose of PZQ. In addition, people self-referred to rural health units are routinely examined for schistosomiasis, and there is no charge for the diagnosis and treatment of schistosomiasis. Note that in Egypt, there is a well-developed network of rural health units, providing primary health care to major as well as satellite villages in the high-density Nile Delta and Nile valley, where almost everyone lives within 5 km of a rural health unit.^8^ Therefore, this approach could readily be used to apply the “5T” strategy in other *S. mansoni*–endemic governorates.

Assessment of potential water contact sites indicated that a considerable number of people (46.2–70.6%) are in contact with potential transmission sites because of occupational activities such as farming in irrigated agricultural areas and fishing. Other water contact activities included domestic activities such as washing clothes and utensils (15.5–39.2%) and recreational activities and swimming (12.6–15.5%). Such observations could explain our finding that assessment of potential water contact sites resulted in identification of the largest number of PICs. Schistosomiasis is a mainly rural, often occupational disease that principally affects people who are unable to avoid contact with water, regardless of the weather.^9^ Note that the current study was conducted during the months of March–June when the average temperature was 20.0°C (68.0°F). If the study had been carried out during warmer or hot months, we may have found more people in contact with potential water contact sites, especially for playing and swimming. Consequently, our study validates assessment of potential water contact sites as a potential approach for implementing the “5T” strategy in other similar endemic areas.

The WHO recommends MDA for control/elimination of several NTDs.^3^ However, cost-effectiveness and achieving and maintaining an optimal high MDA coverage will remain challenges for successful MDA campaigns because infected cases remain unknown. The 5T strategy has prominent advantages over MDA, especially when the prevalence has decreased to low or very low levels. It targets infected individuals detected in the general population for treatment and directly approaches noncompliant individuals or those who are not commonly treated through MDA. In the present study, the compliance for treatment and follow-up after PZQ treatment was excellent, as 99.2% of the infected individuals were treated and 94.8% tracked for a posttreatment follow-up. In other types of longitudinal studies, lower compliance for follow-up after treatment was reported. For example, in two cohort studies, 82.6% and 60.7% of those enrolled were followed up 3–4 weeks^10^ and 1 year after PZQ treatment, respectively.^11^ Such high compliance with the follow-up after treatment could be because the follow-up was carried out after a relatively short period (3–4 weeks) posttreatment. Furthermore, the technicians and health workers who carried out the study are well known to the studied communities which would ensure high-level community participation.

In the present study, we used PZQ at the WHO-recommended dose (40 mg/kg) for treatment of infected subjects in all ages. Note that most of these infected subjects were previously treated with PZQ at least once. However, 14 treatment-naive children (age ≤ 6 years) were treated for the first time. High cure rates (81.7% after one dose and 100% after the second dose) were observed using a sensitive diagnostic test (POC-CCA). These are higher than cure rates (42–79% after single dose and 69–91% after two doses) reported by a review article based on positive to negative conversion in egg detection assays.^12^ It is of special interest that the POC-CCA trace reading was not observed in any of the posttreatment evaluations.^6^

Our observation that the number of identified infected subjects decreased as the number of detection cycles increased is of special interest. Initially, there were 253 PICs, which decreased to 118 SICs (after the second detection cycle), then decreased to 19 seconds SICs (after a third detection cycle). This observation is consistent for the three study villages. Considering that the study villages have a very low *S. mansoni* infection prevalence (≤ 10% by POC-CCA assay), such an observation clearly indicates that these villages are approaching elimination of transmission.

Our findings of the assessment of the “5T” process indicate its feasibility. The process goals are achievable, acceptable to field technicians as well as village dwellers, and probably cost-effective as programs move toward elimination. This approach is certainly cost-effective in regard to drug costs. Note that this is a limited study, and so there was no broad community mobilization. It is worth mentioning that we were not able to find any published formal study based on testing and treating of schistosomiasis with which we could compare of our findings. However, the “5T” as implemented in the present study is currently labor-intensive, and for widespread application, the strategy needs to be streamlined to reduce person-time requirements. For adopting and implementing this strategy on a wide scale by an elimination program, several important issues need to be resolved or fulfilled. First, strong political commitment of the Ministry of Health and Population senior officials, as well as local officials, is a crucial element for its success, as seen in other public health programs.^13^ Second, good advocacy using different venues including advertising posters in schools, rural health units, and agricultural extension stations, and meetings with the chief and religious leaders of target villages would likely help maximize case detection yields. Third, extensive training of field-workers and adequate supervision are essential ingredients for success.
